# Environmental Epigenetics and a Unified Theory of the Molecular Aspects of Evolution: A Neo-Lamarckian Concept that Facilitates Neo-Darwinian Evolution

**DOI:** 10.1093/gbe/evv073

**Published:** 2015-04-26

**Authors:** Michael K. Skinner

**Affiliations:** Center for Reproductive Biology, School of Biological Sciences, Washington State University

**Keywords:** epigenetics, Lamarck, Darwin, natural selection, environment, review

## Abstract

Environment has a critical role in the natural selection process for Darwinian evolution. The primary molecular component currently considered for neo-Darwinian evolution involves genetic alterations and random mutations that generate the phenotypic variation required for natural selection to act. The vast majority of environmental factors cannot directly alter DNA sequence. Epigenetic mechanisms directly regulate genetic processes and can be dramatically altered by environmental factors. Therefore, environmental epigenetics provides a molecular mechanism to directly alter phenotypic variation generationally. Lamarck proposed in 1802 the concept that environment can directly alter phenotype in a heritable manner. Environmental epigenetics and epigenetic transgenerational inheritance provide molecular mechanisms for this process. Therefore, environment can on a molecular level influence the phenotypic variation directly. The ability of environmental epigenetics to alter phenotypic and genotypic variation directly can significantly impact natural selection. Neo-Lamarckian concept can facilitate neo-Darwinian evolution. A unified theory of evolution is presented to describe the integration of environmental epigenetic and genetic aspects of evolution.

## Introduction

**Fig. 1.—:**
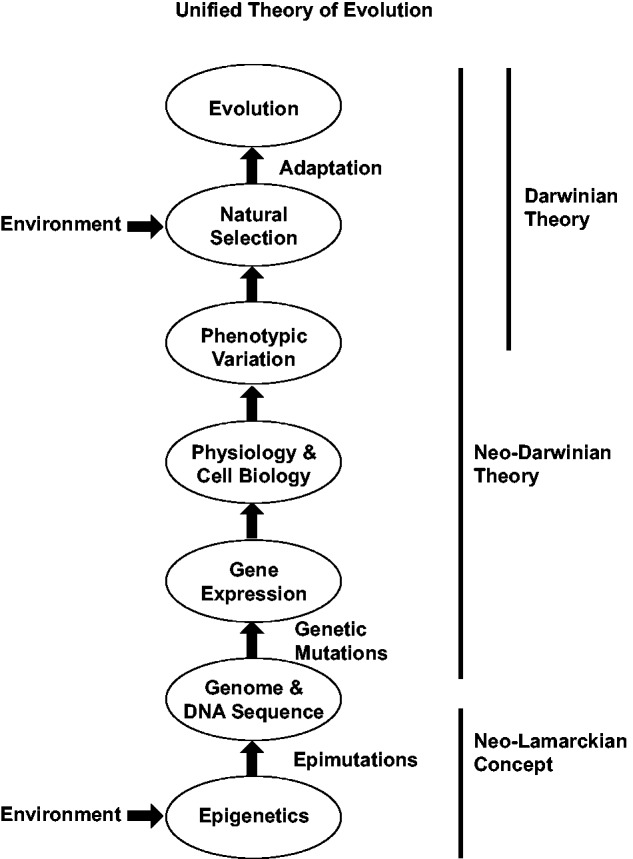
Schematic of the unified theory of evolution. No dominance is suggested by the appearance of specific circles (e.g., epimutations vs. genetics) such that all are equally important components.

Charles Darwin’s concept of evolution by natural selection is the unifying theme for much of modern biology (Darwin 1859). Remarkably, Darwin had no understanding of the molecular mechanisms involved in this process. Integration of Darwin’s thinking with advances in genetic and molecular sciences over the past century facilitated the development of a well supported neo-Darwinian theory of evolution (Olson-Manning et al. 2012). The current primary concept for the molecular basis of evolution involves genetics and mutations, such that random DNA sequence and chromosomal alterations create a genetic variation that directly impacts phenotype and phenotypic variation. The majority of models in evolutionary biology involves DNA sequence mutations as the primary molecular mechanism underlying heritable phenotypic variation (Laland et al. 2014). A conundrum in evolutionary theory is that the frequency of potentially advantageous genetic mutations is extremely low (Jablonka and Raz 2009; Day and Bonduriansky 2011; Kuzawa and Thayer 2011; Nei and Nozawa 2011; Laland et al. 2014). Although recent studies with organisms such as microbes demonstrate genotypic variation are sufficient (Levy and Siegal 2008; Avelar et al. 2013; Ho and Zhang 2014) and additional mechanisms such as random genetic drift, genetic assimilation, directed mutations and epistasis also play important roles, genetic theory alone has difficulty explaining some aspects of evolution (Laland et al. 2014). For example, phenotypic mutation rates and genotypic mutation rates are dramatically different and genetics has been the primary molecular mechanism considered (Burger et al. 2006), but the inclusion of an additional mechanism such as epigenetics can help explain this discordance. Understanding the origins of genotypic variation and rapid evolutionary phenomenon under environmental pressure is difficult to explain with only classic genetics considered. Opposing groups of evolutionary biologists are now debating the need to “rethink” the theory (Laland et al. 2014). Genetics is the primary molecular mechanism considered in classic neo-Darwinian evolution theory (Olson-Manning et al. 2012) (table 1 and fig. 1).

**Table 1:** Evolution Theory Components

Neo-Lamarckian concept
Environment directly alters phenotype generationally
Darwinian evolution theory
Natural selection acts on phenotypic variation
Neo-Darwinian evolution theory
Genetic mutations promote phenotypic variation on which natural selection acts
Unified evolution theory
Environmental epigenetic alterations promote genetic mutations to alter genotypic variation Environmental epigenetics and genetic mutations both promote phenotypic variation on which natural selection acts

In addition to evolution considerations, a large number of biological phenomena have been observed that cannot be easily explained by genetics alone. These include the fact that identical twins with similar genetics generally have discordant disease (Zwijnenburg et al. 2010; Kratz et al. 2014; Tan et al. 2015), or the fact that generally only a small percentage of a disease population has been found to have a correlated genetic mutation, or the fact that many diseases have increased in frequency an order of magnitude in only a couple decades, or the fact that hundreds of environmental contaminants not able to alter DNA sequence have been shown to alter disease or phenotype later in life (Skinner 2014a). Many biological observations do not follow normal Mendelian genetic rules and are difficult to explain with classic genetic processes or mechanisms (McClintock 1984). An example in evolution is that the rates of molecular and morphological evolution are largely decoupled and these patterns of phenotypic divergence are regulatory and not classic genetic mutations (Janecka et al. 2012). Epigenetic resolution of the “curse of complexity” in adaptive evolution of complex traits has been suggested (Badyaev 2014).

Recently documented molecular mechanisms that can dramatically influence genome activity and contribute to phenotypic variation involve epigenetics (Skinner et al. 2010). Many of the above phenomenon when epigenetics is considered as an additional molecular mechanism can be more easily understood, such as the discordance of identical twins (Zwijnenburg et al. 2010; Kratz et al. 2014; Tan et al. 2015). Waddington (1953) coined the term epigenetics and the classic epigenetic definitions of Waddington (1953) and others (Skinner 2011) are descriptive, without an understanding of the molecular elements (Skinner 2011). Considering our current molecular understanding, epigenetics is defined as “molecular processes around DNA that regulate genome activity independent of DNA sequence and are mitotically stable” (Skinner et al. 2010). These epigenetic mechanisms include DNA methylation, histone modifications, chromatin structure, and selected noncoding RNA (ncRNA) (Skinner 2014a). Epigenetic processes such as DNA methylation can become programmed (e.g., imprinted) and be inherited over generations (Skinner 2014a). Environmental factors have been shown to promote the epigenetic transgenerational inheritance of phenotypic variation. Several examples of environmentally induced epigenetic transgenerational inheritance of phenotypic change have been shown to be inherited for hundreds of generations (Cubas et al. 1999). Therefore, like genetic changes, epigenetic changes can have an important role in short-term microevolution (Day and Bonduriansky 2011) and contribute to macroevolutionary (i.e., at or above the level of species) processes, such as speciation and adaptive radiation (Rebollo et al. 2010; Flatscher et al. 2012). A number of insightful reviews have proposed a role for epigenetics in evolution, primarily as a responsive molecular mechanism in natural selection (Jablonka et al. 1998; Pigliucci 2007; Laland et al. 2014).

## Environment and Evolution

A variety of environmental factors can influence evolution and general biology. These range from ecological parameters such as temperature and light to nutritional parameters such as caloric restriction or high fat diets. A host of environmental chemicals from phytochemicals to toxicants can also influence phenotype and health (Skinner 2014a). Environment has a critical role in natural selection and Darwinian evolution (Darwin 1859). Natural selection is a process in which environmental factors influence the survival or reproductive success of individuals bearing different phenotypes. The current paradigm in evolutionary biology holds that changes in DNA sequence underlie the variation that can evolve in response to natural selection (Laland et al. 2014) (table 1). Although James Baldwin in 1896 suggested environment through sociobiology type mechanisms (i.e., behavior) could alter phenotypic variation, these are thought to be due to genetic changes and considered a neo-Darwinian process (Baldwin 1896; Paenke et al. 2007). Therefore, in neo-Darwinian evolution the primary link between the environment and evolution is to mediate the natural selection process (Olson-Manning et al. 2012; Laland et al. 2014).

In contrast, Lamarck proposed one of the early evolutionary theories in 1802 in that environment promotes the phenotypic alterations associated with evolution (Lamarck 1802; Calabi 2001). This is distinct to the role of environment providing selective pressure in natural selection, such that environment directly alters the phenotype to influence evolution. This theory was seen as conflicting with Darwin’s natural selection evolutionary theory and so was discounted and today is not seriously considered in modern evolutionary theory or neo-Darwinian evolution (Day and Bonduriansky 2011). However, if there was a molecular mechanism that generationally could facilitate the ability of the environment to alter genotypic and phenotypic variation, such a neo-Lamarckian concept may facilitate evolution (table 1 and fig. 1).

Interestingly, Darwin (1868) himself was a strong proponent of the inheritance of acquired characteristics. The blending of inheritance and evolution by natural selection appeared to be a fundamentally flawed concept that would require an untenably high mutation rate in order to maintain the trait variation required for selection (Jenkins 1867). To address this, Darwin (1868) proposed pangenesis, a complex theory of environmentally responsive somatic cell transmittance to offspring. Therefore, Darwin conceptually supported Lamarck’s theory of the inheritance of acquired characteristics, but until the last 30 years the potential molecular mechanism was unclear.

## Environmental Epigenetics

Epigenetics provides molecular mechanisms for the environment to directly alter phenotypic variation and its subsequent inheritance (Crews et al. 2007; Skinner, Gurerrero-Bosagna, Haque, et al. 2014). A variety of epigenetic mechanisms have been identified including DNA methylation, histone modifications, chromatin structure, and selected ncRNA. All these mechanisms have the ability to program and alter gene expression and have been shown to have a critical role in normal development and biological processes (Skinner et al. 2010; Skinner 2014a). For example, the ability to generate an embryonic stem cell requires the erasure of DNA methylation such that the cell becomes pluripotent (Seisenberger et al. 2013). Although the vast majority of environmental factors cannot alter DNA sequence, epigenetic processes can be dramatically altered in response to environmental factors from nutrition to temperature (Skinner 2014a). All organisms that have been investigated contain highly conserved epigenetic processes (e.g., DNA methylation) that can be environmentally modified (Skinner 2014a). Epigenetics provides an additional molecular mechanism, integrated with genetics, to regulate biology.

The ability of environment to directly alter the development and function of cells and tissues is critical for the health and phenotype of the individual. This direct environmental epigenetic effect on the individual would likely have a limited impact on evolution, unless the epigenetic changes could be transmitted between generations. A large number of environmental factors from nutrition to toxicants have been shown to induce the epigenetic transgenerational inheritance of disease and phenotypic variation (Skinner 2014a). Epigenetic transgenerational inheritance is defined as the germline transmission of epigenetic information between generations in the absence of direct exposure (Skinner et al. 2010). Environmental exposures during a critical period of germline development, fetal gonadal sex determination or gametogenesis, have been shown to permanently program epigenetic marks such as DNA methylation (Skinner 2014a). Nutrition (Pembrey et al. 2006; Burdge et al. 2011), temperature (Song et al. 2013), stress (Skinner 2014b), and toxicants (Anway et al. 2005; Skinner 2014a) have all been shown to promote the epigenetic transgenerational inheritance of phenotypic variation (Skinner 2014a). The phenomenon has been observed in plants, insects, fish, rodents, pigs, and humans (Skinner 2014a). In mammals the altered transgenerational phenotypes have been observed for generations (Skinner 2014a), with environmentally induced epigenetic transgenerational inheritance of phenotypic variation in plants being transmitted for hundreds of generations (Cubas et al. 1999). Therefore, environment can promote the epigenetic transgenerational inheritance of phenotypic variation. The ability of environment to alter phenotype and alter phenotypic variation, independent of genetics, through this epigenetic mechanism is proposed to be important for evolution (Anway et al. 2005; Jablonka and Raz 2009; Day and Bonduriansky 2011; Kuzawa and Thayer 2011; Skinner 2014a).

Darwin proposed that one of the critical determinants of evolution was sexual selection (Darwin 1859). A previous study investigated the ability of an environmental factor (toxicant) to promote the epigenetic transgenerational inheritance of an alteration in mate preference associated with sexual selection (Crews et al. 2007). An F0 generation gestating female rat was exposed to the agricultural fungicide vinclozolin transiently and then the F3 generation animals (great-grand-offspring) were obtained to assess alterations in mate preference behavior (Anway et al. 2005). A dramatic alteration in mate preference was observed (Crews et al. 2007) along with epigenetic alterations (termed epimutations) in the germline (sperm) (Guerrero-Bosagna et al. 2010). Transgenerational transcriptome changes in the brain regions correlated with the alterations in mate preference behavior (Skinner et al. 2008). Therefore, an environmental factor that altered sexual selection was found to promote a permanent alteration in the sperm epigenome in an imprinted-like manner that was inherited for multiple generations (Crews et al. 2007; Skinner et al. 2010). These studies suggest that environmental epigenetics may play an important role in evolutionary change. The role of epigenetics in mate choice and evolution has been further discussed (Zeh JA and Zeh DW 2008; Bonduriansky and Day 2013). Indeed, several recent reviews have suggested a role for epigenetics in microevolution and macroevolution (Jablonka and Raz 2009; Rebollo et al. 2010; Skinner et al. 2010; Day and Bonduriansky 2011; Kuzawa and Thayer 2011; Flatscher et al. 2012; Klironomos et al. 2013; Badyaev 2014; Jaeger and Monk 2014; Skinner 2014a).

## Unified Theory

Environmental epigenetics and epigenetic transgenerational inheritance provide a molecular mechanism for the neo-Lamarckian concept that environmental factors directly alter phenotype (table 1). The ability of environmental epigenetics to alter phenotypic variation provides an initial element for evolution where environment can directly establish the variation and phenotype in a population (fig. 1). Although aspects of the original Lamarckian evolution theory were not accurate (Lamarck 1802), such as having “directed” phenotypes within a generation (Koonin and Wolf 2009; Koonin 2014), the concept that environment can directly impact phenotype is supported by environmental and transgenerational epigenetic studies (Crews et al. 2007; Koonin and Wolf 2009; Koonin 2014; Skinner, Gurerrero-Bosagna, Haque, et al. 2014). Therefore, the first aspect of the unified theory involves the ability of environment to impact epigenetic programming generationally to alter phenotypic variation (fig. 1).

The well-established aspect of Darwinian evolution is the ability of environment through natural selection to act on phenotypic variation within an evolutionary event (Darwin 1859; Olson-Manning et al. 2012). The classic neo-Darwinian view is that genetic mutations and genetic variation are the primary molecular mechanism involved in generating the phenotypic variation (Nei and Nozawa 2011; Olson-Manning et al. 2012) (table 1). Although epigenetics can also have a critical role in the establishment and maintenance of phenotypic variation, the genetic mutations and genotype of the phenotype will be critical. This neo-Darwinian natural selection event for evolution is the other component of the unified theory (fig. 1).

A combination of environmental epigenetic impacts on phenotypic variation and the ability of environment to mediate natural selection will both be important for evolution. Therefore, this neo-Lamarckian concept facilitates neo-Darwinian evolution (fig. 1). This unified theory provides an expanded understanding of the molecular aspects of evolution and solutions for issues such as the mechanisms for rapid evolutionary phenomenon. The mechanisms that environment can impact evolution are also expanded. An integration of epigenetics and genetics will be essential to consider in our future understanding of the molecular aspects of evolution (Jablonka and Raz 2009; Day and Bonduriansky 2011; Laland et al. 2014; Skinner 2014a).

An additional important consideration involves the ability of epigenetic processes to promote genetic mutations (table 1). In cancer biology, altered epigenetics has been shown to promote genome instability and formation of genetic mutations (Feinberg 2004). Nearly all genetic mutations can be directly influenced by epigenetic processes. The most frequent point mutation (single nucleotide polymorphism) is a C to T conversion that is facilitated by CpG DNA methylation (Jones et al. 1992). Repeat elements in the genome when expanded create copy number variations (CNV) that are controlled by hypermethylation of DNA (Liu et al. 2012). Transposable elements are also silenced by hypermethylation of DNA (Yagi et al. 2012). Translocation events and inversions are also influenced by histone modifications, DNA methylation, and ncRNA (Solary et al. 2014). Therefore, epigenetics can directly influence genetic mutations and the origin of genotypic variation is influenced by environmental epigenetic alterations (table 1). In contrast, genetic mutations have been shown to influence epigenetics (Furey and Sethupathy 2013). Recently, we have found that environmentally induced epigenetic transgenerational inheritance of disease and phenotypic variation can promote genetic mutations (i.e., CNV) in later generations (Skinner MK, Guerrero-Bosagna C, Haque MM, unpublished data). Therefore, environmental epigenetics may not only promote increased phenotypic variation, but epigenetics can also drive genetic change and increase genotypic variation. This also needs to be considered in the unified evolution theory (fig. 1).

## Discussion

Environmental epigenetics and epigenetic transgenerational inheritance alter phenotypic variation which can be acted on by natural selection. Therefore, environmental epigenetics can directly influence phenotype and this neo-Lamarckian concept can facilitate natural selection and neo-Darwinian evolution. These different aspects of evolution should not be seen as conflicting, but instead can form a unified theory for evolution (fig. 1). This expanded understanding of the molecular aspects of evolution provides novel insights into the mechanism for rapid evolutionary events. An expanded understanding of how environment impacts evolution is also provided. The unified theory provides novel considerations that environment can both act to directly influence phenotypic variation and directly facilitate natural selection (fig. 1). Previous evolutionary models have primarily considered genetics and mutations as the primary molecular driver for evolution (Nei and Nozawa 2011; Olson-Manning et al. 2012; Laland et al. 2014). More recently, a number of models have started to consider epigenetics in these evolution models as well (Rebollo et al. 2010; Skinner et al. 2010; Day and Bonduriansky 2011; Kuzawa and Thayer 2011; Flatscher et al. 2012; Klironomos et al. 2013; Badyaev 2014; Jablonka and Lamb 2014; Jaeger and Monk 2014). For example, consideration of epigenetics as an additional molecular mechanism has assisted in the understanding of genetic drift (Gordon et al. 2012), genetic assimilation (Zuckerkandl and Cavalli 2007), and directed mutation (Jablonka and Lamb 2007; Kryazhimskiy et al. 2014). The consideration of epigenetics can also be used to better understand neutral evolution (Kimura 1989) through mechanisms, such as robustness (Ohta 2011). The unified theory suggests additional variables that should be considered are the multiple roles of environment and the integration of epigenetics into future evolution models.

Epigenetic transgenerational inheritance of phenotypic variation will have an important role in microevolutionary and macroevolutionary changes, including speciation. A recent study was designed to investigate the epigenetic changes associated with phylogenetic distance in Darwin’s finches (Skinner, Gurerrero-Bosagna, Haque, et al. 2014), a well-known example of adaptive radiation (Darwin 1859; Lack 1947; Burns et al. 2002; Grant and Grant 2008; Huber et al. 2010; Donohue 2011). Erythrocyte DNA was obtained from five species of sympatric Darwin’s finches that vary in phylogenetic relatedness. Genome-wide alterations in genetic mutations, using CNV, were compared with epigenetic alterations associated with differential DNA methylation regions (epimutations) (Skinner, Gurerrero-Bosagna, Haque, et al. 2014). A greater number of epimutations than genetic mutations were observed among the different species, with the number of epimutations increasing with phylogenetic distance. The number, chromosomal locations, regional clustering, and overlap of epimutations suggest that epigenetic change has likely had a role in the speciation and evolution of Darwin’s finches (Skinner, Gurerrero-Bosagna, Haque, et al. 2014). A number of additional observations also support a role of epigenetics and speciation. Using *Drosophila* and maternally inherited ncRNA silencing of transposons a role for epigenetics and speciation was discussed (Brennecke et al. 2008). The role of epigenetics and a punctuated equilibrium in the mobilization of transposable elements was also suggested (Zeh et al. 2009). An interesting study comparing Neanderthal and human DNA methylation maps also supports a role for epigenetics in speciation (Gokhman et al. 2014) and evolution.

Although the causal role of epimutations was not established in the Darwin’s finch adaptive radiation (Skinner, Gurerrero-Bosagna, Haque, et al. 2014) or other models (Brennecke et al. 2008; Zeh et al. 2009; Gokhman et al. 2014), the causal role of genome-wide genetic mutations has also not been established (Laland et al. 2011). Future studies need to focus on the causal relationship of epigenetic alterations in relation to phenotypic variation that is acted on by natural selection. Genetics and genetic mutations are critical for evolution, but they are not the only molecular factors to consider. Although the major paradigm in the biological sciences is genetic determinism, this paradigm is limited in its ability to explain biological phenomenon ranging from the molecular basis of disease etiology (Skinner 2014a) to certain aspects of evolution by natural selection (Skinner et al. 2010; Day and Bonduriansky 2011; Longo et al. 2012). As Thomas Kuhn suggested during a scientific revolution when the current paradigm reveals anomalies then new science needs to be considered (Kuhn 1962). This type of challenge to current paradigms is also supported by other scientific philosophy, such as Popper (Rieppel 2008) and Macintyre (MacIntyre 1977). A paradigm shift is required to explain how genetics and epigenetics integrate to regulate genome activity and evolution, and these advances will need to be incorporated into future evolutionary biology modeling (Rebollo et al. 2010; Skinner et al. 2010; Day and Bonduriansky 2011; Kuzawa and Thayer 2011; Flatscher et al. 2012; Klironomos et al. 2013; Badyaev 2014; Jablonka and Lamb 2014; Jaeger and Monk 2014; Skinner 2014a) and theory.

## Summary

The integration of environmental epigenetics into the molecular aspects of evolution theory suggests a neo-Lamarckian concept that facilitates neo-Darwinian evolution. Several of the novel factors to be considered are summarized below. In regards to the neo-Lamarckian concept:

1. Environmental epigenetics provides a molecular mechanism for Lamarck’s proposal that environment can directly alter phenotype in a heritable manner.
2. Environmental exposures at critical developmental windows promote the epigenetic transgenerational inheritance of germline (e.g., sperm) epimutations that alter phenotypic variation.
3. Direct environmental exposures of developing somatic tissue can alter somatic epigenomes and phenotype in the individual exposed, but this will not be heritable and the phenotypes will often be distinct to transgenerational phenotypes.
4. In regards to novel aspects of neo-Darwinian evolution:
5. Transgenerational germline epimutations alter genome stability to promote genetic mutations and genotypic variation in subsequent generations.
6. Phenotypic variation is derived from a combination of integrated genetic and epigenetic processes on which natural selection acts.
7. Environment has a critical role in natural selection, as well as in the induction of heritable adaptive phenotypic variation.

As shown in figure 1, these concepts and components contribute to a unified theory that integrates environmental epigenetics into the molecular aspects of evolution. It is important to note that there is not a dominance of genetics or epigenetics, but the two molecular processes integrate to regulate biology.

Previously, an environmental exposure was found to promote the epigenetic transgenerational inheritance of phenotypic traits such as mate preference, which can play an important role in evolution (Crews et al. 2007; Skinner 2014a). Several reviews have subsequently suggested a role for epigenetics in evolution (Jablonka and Raz 2009; Rebollo et al. 2010; Skinner et al. 2010; Day and Bonduriansky 2011; Kuzawa and Thayer 2011; Flatscher et al. 2012) and experimental models have shown the importance of epigenetic associated genes (Mihola et al. 2009) and molecular elements (Long et al. 2013; Skinner, Gurerrero-Bosagna, Haque, et al. 2014) in evolution. The current report extends these studies to present a unified theory that combines both neo-Lamarckian and neo-Darwinian aspects and expands our understanding of how environment impacts evolution. The integration of epigenetics and genetics will be critical for all areas of biology including evolution.
